# Age of smoking initiation and risk of breast cancer in a sample of Ontario women

**DOI:** 10.1186/1617-9625-5-4

**Published:** 2009-02-17

**Authors:** Erin Young, Scott Leatherdale, Margaret Sloan, Nancy Kreiger, Andriana Barisic

**Affiliations:** 1 Dalla Lana School of Public Health, University of Toronto, Toronto, Ontario, Canada; 2 Department of Population Studies and Surveillance, Cancer Care Ontario, Toronto, Ontario, Canada; 3 Department of Health Studies and Gerontology, University of Waterloo, Waterloo, Ontario, Canada; 4 Department of Nutritional Sciences, University of Toronto, Toronto, Ontario, Canada

## Abstract

**Objectives:**

To examine the association between time of smoking initiation and both the independent and joint effects of active and passive tobacco smoke exposure and the risk of breast cancer in a sample of Ontario women.

**Methods:**

Data from two large population-based case-control studies conducted among Ontario women aged 25–75 years were combined for analysis (n = 12,768).

**Results:**

Women who had ever smoked and were exposed to passive smoke had a significant increased risk of breast cancer (OR 1.13, 95%CI 1.01–1.25). A significant increased risk was also observed among women who initiated smoking: at age 26 or older (OR 1.26, 95%CI 1.03–1.55); more than five years from menarche (OR 1.26, 95%CI 1.12–1.42); and, after their first live birth (OR 1.25, 95%CI 1.02–1.52).

**Conclusion:**

The results suggest that women who initiate smoking at an older age are at an increased risk of breast cancer.

## Introduction

Smoking is the largest preventable cause of cancer [1], yet smoking rates among women remain high. In 2007, 18.1% of Canadian women aged 15 and older were current smokers [2], with young adult females aged 20–24 exhibiting the highest prevalence of use (25.6%). Considering that declines in the prevalence of smoking among young adult females have reached a plateau [2], and older ages of smoking onset among young adult women is an emerging trend [3], it appears that smoking will continue to represent a considerable public health burden among Canadian women.

Breast cancer, the most common cancer in Canadian women excluding non-melanoma skin cancer, is a leading cause of cancer-related morbidity and mortality in Canada [4]. There is some evidence of a link between tobacco smoke exposure and breast cancer [e.g., [1,5-16]]. Animal models have demonstrated that mammary tissue may have increased susceptibility to carcinogenic exposures during the years from pre-puberty to age at first full-term pregnancy [17,18]. The breast experiences its highest rate of cellular proliferation during this period and there may be a decreased ability of DNA repair mechanisms to correct damage before cell division occurs [6]. This is consistent with the hypothesis that cigarette smoke, either active or passive, in this age period increases the risk of breast cancer in women. However, the majority of studies over the past few decades examining smoking during this age period have produced inconsistent results [6-16]. In order to help clarify this relationship, we examined the association between breast cancer and active and passive smoking both independently and jointly in a combined data set of two case-control studies conducted in Ontario.

## Methods

Data from the Ontario Women's Health Study (OWHS) (conducted between June 1996 and May 1998 among 6195 women aged 25 to 75 years [19]), and the Ontario Women's Diet and Health Study (OWDHS) (conducted between June 2002 and April 2003 among 6573 women aged 25 to 75 years [20]) were combined for this secondary analysis. The OWDHS and OWHS are large population based-based-case-control studies. Both had similar target populations, data collection methodologies, and measures. Ethics approval was granted by the University of Toronto Research Ethics Board [19,20].

### Cases

Women age 25–74 years and who were diagnosed with a first, pathologically confirmed cancer of the breast were identified in both studies through the Ontario Cancer Registry (OCR). The OCR is a population-based cancer registry and includes cases of invasive cancer diagnosed among residents of Ontario [21]. Over 95% of all breast cancer pathology reports from the hospitals and laboratories in Ontario are received by the OCR, all nearly within three months of biopsy. Due to the high number of breast cancer cases diagnosed each year in Ontario (~4400), the OWHS sampled cases randomly using a 50% sampling fraction, while the OWDHS selected all cases in an 11-month period. Pathology reports were used to identify physicians to obtain consent to contact and contact information for the cases. Physician cooperation was high for both studies, 95% in the OWHS and 96% in the OWDHS. Physician response was over 90%.

### Controls

Controls for both studies were age-stratified random samples of Ontario women who were 1:1 frequency matched to cases within five-year age groups. The OWHS identified controls in the 1996 population-based assessment rolls database of the Ontario Ministry of Finance (MOF), which included full name, age, sex, and address for all homeowners and tenants in Ontario. The OWDHS identified controls through random digit dialing.

### Data collection

Both the OWHS and the OWDHS used self-administered mailed questionnaires containing questions regarding known or suspected risk factors for breast cancer including various measures of active and passive tobacco smoke exposures. Both studies made efforts to improve response by including a five dollar incentive [22], and by sending non-responders a reminder post-card after two weeks, followed by a telephone call after four weeks. The response rate for the OWHS, after deleting cases for whom physician consent was not obtained, was 73% (3133 of 4289 women) for cases and 61% (3062 of 5001 women) for controls. The response rate for the OWDHS was 75% (3102 of 4109 women) for the cases and 85% (3471 of 4102 eligible women) for the controls. The difference in the response rate for the controls among the two studies is attributable to the control selection method in the OWDHS, where only those women who expressed some interest in participating in the study when contacted by random digit dialing were mailed a questionnaire.

### Definitions of exposure

Consistent with the Canadian Tobacco Use Monitoring Survey (2007) [2], ever active smoking was conservatively defined as having ever smoked at least 100 cigarettes in their lifetime. Participants were also asked how many hours in a day they were exposed to the tobacco smoke of others as a child and approximately two years ago (the latter for both working and non-working days). Those who were exposed to less than 2 hours a day on average were categorized as having no passive smoke exposure and those who were exposed to 2 or more hours per day in childhood, adulthood or both were considered to be exposed to passive smoke. The referent category throughout the analysis was defined as no history of active or passive smoking. Although passive smoke exposure and active smoke exposure were conservatively defined (due to the limitations of the measures available), using such conservative estimates biases our results to the null, consequently any significant associations likely represent meaningful differences across groups.

### Statistical analysis

A total of 6235 cases and 6533 controls were available for this analysis. Women were excluded if they had missing data for age at menarche (n = 332); the analysis examining age at smoking initiation in relation to first live birth included only parous women (n = 11039). Logistic regression analyses were used to examine the risk of breast cancer in relation to smoking exposure, age of initiation, smoking initiation in relation to menarche, and smoking initiation in relation to first live birth. A category for missing values for each variable was included in all models and adjustments were made for the frequency matching variable age and for other potential confounders: age of menarche, age of first live birth, parity, BMI, oral contraceptive use, hormone-replacement therapy, alcohol consumption, menopausal status, family history of breast cancer, history of benign breast disease and household income. None of the potential confounders changed the risk estimates by more than 10%; as such, only age-adjusted odds ratios are presented. All analyses were performed with SAS version 9.1 [23].

## Results

Since the distribution of selected subject characteristics and risk factors for cases and controls were similar across the two studies (data not shown), the combined descriptive data are presented in Table 1. In general, the distributions within our sample are consistent with existing research, in which cases had a significantly increased risk of breast cancer with use of hormone replacement therapy, family history of breast cancer, history of benign breast disease, early age of menarche, older age at first birth, and nullparity [6-16].

**Table 1:** Distribution of demographic variables and known breast cancer risk factors among women in the combined studies

**Risk Factor**	**Cases** **(n = 6065)**		**Controls** **(n = 6371)**		
	**No.**	**%**	**No.**	**%**	**Chi-Square**
**Age**					
25 – 34	118	1.9	200	3.1	χ^2 ^= 43.21, df = 4, p < .001
35 – 44	807	13.3	1001	15.7	
45 – 54	1739	28.7	1868	29.3	
55 – 64	1823	30.1	1704	26.8	
65 – 75	1578	26.0	1598	25.1	
**Age at menarche**					
< 12	1183	19.5	1155	18.1	χ^2 ^= 10.90, df = 2, p < .01
12 – 14	4200	69.3	4389	68.9	
15 +	682	11.2	827	13.0	
**Menopausal status**					
Pre	1784	29.4	2133	33.5	χ^2 ^= 22.15, df = 3, p < .001
Post	4192	69.1	4184	65.1	
Don't Know	85	1.4	84	1.3	
Missing	4	0.1	6	0.1	
**Pregnancy**					
Never	773	12.7	603	9.5	χ^2 ^= 33.98, df = 2, p < .001
Ever	5282	87.1	5757	90.4	
Missing	10	0.2	11	0.2	
**Age at first live birth**					
Null parous	773	12.8	603	9.5	χ^2 ^= 62.00, df = 5, p < .001
< 21	1141	18.8	1321	20.7	
21 – 24	1545	25.5	1870	29.4	
25 – 29	1570	25.9	1612	25.3	
30 +	807	13.3	772	12.1	
Missing	229	3.8	193	3.0	
**Number of live births**					
Null parous	773	12.6	603	9.5	χ^2 ^= 69.84, df = 4, p < .001
1 child	771	12.7	755	11.9	
2 children	2197	36.2	2255	35.4	
3 + children	2102	34.7	2584	40.6	
Missing	222	3.7	174	2.7	
**Use of oral contraceptives**					
No	2815	46.4	2825	44.3	χ^2 ^= 11.54, df = 3, p < .01
Yes	3184	52.5	3466	54.4	
Don't Know	46	0.8	40	0.6	
Missing	20	0.3	40	0.6	
**Use of hormone replacement therapy**					
No	3954	65.2	4328	67.9	χ^2 ^= 15.32, df = 3, p < .01
Yes	2048	33.8	1994	31.3	
Don't Know	37	0.6	20	0.3	
Missing	26	0.4	29	0.5	
**Family history of breast cancer**					
No	4692	77.4	5367	84.2	χ^2 ^= 141.53, df = 3, p < .001
Yes	1130	18.6	707	11.1	
Don't Know	65	1.1	69	1.1	
Missing	178	2.9	228	3.6	
**Benign breast disease**					
No	3227	53.2	4688	73.6	χ^2 ^= 565.80, df = 3, p < .001
Yes	2577	42.5	1482	23.3	
Don't know	58	1.0	42	0.7	
Missing	203	3.3	159	2.5	
**Body mass index **(kg/m^2^)					
< 18.5	91	1.5	110	1.7	χ^2 ^= 8.64, df = 5, p = .124
18.5 – 24.9	2573	42.4	2836	44.5	
25 – 29.9	2028	33.4	2091	32.8	
30 – 34.9	861	14.2	832	13.1	
35+	435	7.2	425	6.7	
Missing	77	1.3	77	1.2	
**Family income adequacy**					
Low	663	10.9	725	11.4	χ^2 ^= 6.11, df = 4, p = .191
Middle	2033	33.5	2215	34.8	
High	2183	36.0	2268	35.6	
No answer	1007	16.6	1006	15.8	
Missing	179	2.9	157	2.5	
**Alcohol use **(drinks/week)					
Never drink	1339	22.1	1358	21.3	χ^2 ^= 17.18, df = 5, p < .01
< 1	1277	21.1	1451	22.8	
1 – 3	1796	29.6	1980	31.1	
4 – 6	630	10.4	616	9.7	
7 +	939	15.5	866	13.6	
Missing	84	1.4	100	1.6	

The frequency distributions and the age-adjusted odds ratios for breast cancer in relation to smoking status and timing of smoking initiation are presented in Table 2. Notable significant findings include: women who were active smokers and exposed to passive smoke had increased risk of breast cancer (OR 1.13, 95%CI 1.01–1.25) compared to women with no history of active or passive smoking; there was a monotonic increase in the risk of breast cancer in relation to increasing age of smoking initiation; women who began smoking at age 26 or older had increased risk of breast cancer (OR 1.26, 95%CI 1.03–1.55) compared to women with no history of active or passive smoking; women who initiated smoking more than 5 years after menarche had increased risk of breast cancer (OR 1.26, 95%CI 1.12–1.42) compared to women with no history of active or passive smoking; women who initiated smoking after their first live birth were at increased risk for breast cancer (OR 1.25, 95%CI 1.02–1.52) compared to women with no history of active or passive smoking; and, women who initiated smoking more than five years before their first live birth were at increased risk for breast cancer (OR 1.16, 95%CI 1.04–1.31) compared to women with no history of active or passive smoking.

**Table 2:** Distribution of cases and controls and age-adjusted odds ratios for breast cancer among women in the combined studies in relation to smoking status and time of active smoking initiation

	Cases (n = 6065*)		Controls (n = 6371*)		
Risk Factor	No.	%	No.	%	AOR (95% CI)^†^
Smoking exposure					
Never active/never passive	1270	24.2	1398	25.1	1.0 (ref)
Passive only	1481	28.2	1699	30.5	0.97 (0.88–1.08)
Active only	1143	21.8	1124	20.2	1.10 (0.98–1.23)
Both Active and Passive	1352	25.8	1343	24.1	1.13 (1.01–1.25)
Age of initiation					
Never active/never passive smoker	1270	29.2	1398	31.7	1.0 (ref)
< 12	44	1.0	58	1.3	0.88 (0.59–1.31)
12 – 15	759	17.8	848	19.2	1.02 (0.90–1.16)
16 – 20	1586	37.2	1563	35.4	1.12 (1.01–1.24)
21 – 25	369	8.7	350	7.9	1.13 (0.96–1.33)
26 +	235	5.5	195	4.4	1.26 (1.03–1.55)
Initiation in relation to menarche					
Never active/never passive smoker	1270	34.0	1368	36.0	1.0 (ref)
Before menarche	216	5.8	283	7.4	0.87 (0.71–1.05)
Initiate ≤ 5 years from menarche	1242	33.2	1290	34.0	1.07 (0.96–1.19)
Initiate > 5 years from menarche	1008	27.0	860	22.6	1.26 (1.12–1.42)
Initiation in relation to first live birth ‡					
Never active/never passive smoker	1087	34.5	1231	36.5	1.0 (ref)
Before first birth:					
> 5 years before	1132	35.9	1130	33.6	1.16 (1.04–1.31)
≤ 5 years before	677	21.5	788	23.4	0.96 (0.84–1.09)
After first birth	254	8.1	219	6.5	1.24 (1.02–1.52)

* Numbers may not add to total due to missing values

^† ^AOR = age adjusted odds ratio: adjusted only for age since no confounders were identified

‡ Only among parous women (n = 11039)

Analyses stratified by age of menarche and by age of first live birth demonstrated the same risk patterns as seen in Table 2 (data not shown). Furthermore, as the age cutoff for early age of menarche was set lower, the odds ratio for breast cancer risk due to smoking before menarche increased, although the results were not significant.

## Discussion

Consistent with reviews of existing research [1,24-26], we identified that women living in Ontario exposed to both active and passive cigarette smoke were at increased risk of breast cancer when compared to never active and never passive smokers. We decided to look at passive smoke exposure in women already exposed to active smoke as studies have established a difference in the carcinogenic mechanisms between active and passive smoke exposure on breast cancer risk [27]. Tobacco specific nitrosamines and other carcinogens are more concentrated in passive smoke than mainstream smoke [27]. In addition, in comparison to mainstream smoke, a greater proportion of sidestream smoke is in the vapor phase than in the particulate phase, which results in greater absorption of chemicals into the blood and lymph systems. Therefore it has been suggested that exposure to passive smoke can present a greater risk than active smoke alone [27,28].

This finding may have important implications for cancer control when one considers that smoking rates among Ontario women have declined in the past decade [2], and fewer Ontario women will be exposed to passive smoke due to the new restrictions associated with the 2006 Ontario Tobacco Strategy which banned smoking in public places in Ontario [29]. It may be possible that, even though breast cancer rates in Ontario are currently on the rise [4], such population-level reductions in tobacco smoke exposure (through both active and passive smoking) may have a positive impact on reducing future breast cancer rates in the province.

Conversely, our data suggest that the risk of breast cancer monotonically increased with increased age of smoking initiation. This relationship has been reported in several case-control studies [6,7,14,15] and one cohort study [16]. Although the existing evidence is mixed and other studies have reported an inverse relationship between age of initiation and breast cancer risk [30], such inconsistencies may be attributed to variations in study design and differences in measuring smoke exposure. Similarly, with our conservative estimates we also identified an increased risk of breast cancer among women who initiated smoking after their first live birth and among women who initiated smoking more than five years after menarche. These findings are also consistent with existing studies examining breast cancer risk and timing of smoking around pregnancy [7,12,15,16] and menarche [10,12,14]. Given the emerging trend of older ages of smoking onset among women in Canada [3], the relationship between older ages of smoking onset and breast cancer risk may be cause for concern and requires additional investigation.

### Implications for research and practice

Our findings suggest that there is a need to further examine the impact of population-level shifts in the prevalence of smoking, exposure to passive tobacco smoke, and increasing ages of smoking onset among women on future breast cancer rates. In addition, in 2004, although 89% of 25–34 year old Canadian women reported visiting a doctor, the majority of them reported that they were not asked about their smoking behaviour, and among those who were current smokers, only 39% reported being provided with cessation information from their doctor [31]. Since health professionals can play an important role in reducing smoking uptake and cessation, a simple population-level intervention may involve having doctors systematically ask young adult female patients about their smoking behaviour and provide cessation assistance (e.g., nicotine replacement therapy) to those smokers who want to quit, according to clinical practice guidelines. It has been shown that such smoking cessation interventions are both successful and cost effective [32].

### Limitations

Due to the case-control study design and the use of self-reported questionnaires, there is a possibility of recall bias of their active and passive smoking history, however, research suggests that the impact of recall bias in case-control studies of smoking and breast cancer is limited [15,33]. There is also the possibility of misclassification for the age of smoking initiation, age of menarche and age of first live birth; however, this is unlikely as smoking is not a widely perceived risk factor for breast cancer [10], and certainly not at the time of our data collection. The measures for active and passive smoking are very conservative and likely provide estimates of actual exposure which should bias results to the null. Although more robust measures of smoking (i.e. intensity, puff volume, or pack years) would have been useful, we were restricted due to the limitations of the OWHS and OWDHS questionnaires used for analysis. Moreover, our subjective self-report measures of active and passive smoking would be more robust if validated with objective biochemical analyses of salivary cotinine [34]. At the time these studies were initiated, passive smoking was not recognized as an established risk factor for breast cancer; and as a result the measures used for passive smoke exposure are not as robust as would be ideal. However, the conservative definitions do not necessarily negatively affect our findings as the primary purpose of the study is to examine the risk of breast cancer among women ever exposed to active and passive smoke compared to those never exposed.

We were able to control for passive smoke exposure by including both never active and never passive smokers in the referent group. Although it is possible those exposed to passive smoking were included in the unexposed category, thereby inadvertently diluting the effects of passive smoking; studies with more robust exposure measurements that are better able to limit passive smoke exposure in the referent group tend to demonstrate an even higher breast cancer risk. Therefore, our conservative estimates actually underestimate the association between passive smoke exposure and breast cancer. There were also a large number of missing values, but these were largely confined to the referent group as expected (i.e., cases tend to have higher response rates due to their diagnosis of breast cancer) and were approximately equal among the cases and the controls. The slight differences that did exist are not likely to bias the estimates since there is no reason to expect that the non-response was associated with the exposure of interest (i.e., active and passive smoking).

## Conclusion

The population-level shifts in the prevalence of smoking, exposure to passive tobacco smoke, and increasing ages of smoking onset among women are likely to have an impact on breast cancer rates due to the relationship between breast cancer and active and passive tobacco smoke exposure. As such, practitioners should consider targeting smoking prevention initiatives not only to female youth, but also to young adult female populations.

## Competing interests

The authors declare that they have no competing interests.

## Authors' contributions

EY preformed the analysis, interpretation of the results and the writing of the manuscript. SL and NK oversaw the analysis, interpretation of results, conceptualized the research questions and writing of the manuscript. PS oversaw the analysis and interpretation of the results. AB contributed to the writing of the manuscript.
